# *MAF* Amplification and Adjuvant Clodronate Outcomes in Early-Stage Breast Cancer in NSABP B-34 and Potential Impact on Clinical Practice

**DOI:** 10.1093/jncics/pkab054

**Published:** 2021-05-28

**Authors:** Alexander H G Paterson, Peter C Lucas, Stewart J Anderson, Eleftherios P Mamounas, Adam Brufsky, Luis Baez-Diaz, Karen M King, Thomas Lad, André Robidoux, Melanie Finnigan, Miguel Sampayo, Juan Carlos Tercero, Joël Jean Mairet, Antonio C Wolff, Louis Fehrenbacher, Norman Wolmark, Roger R Gomis

**Affiliations:** NSABP Foundation and NRG Oncology, Pittsburgh, PA, USA; Department of Oncology, University of Calgary, Alberta, Canada; NSABP Foundation and NRG Oncology, Pittsburgh, PA, USA; Department of Pathology, University of Pittsburgh School of Medicine, Pittsburgh, PA, USA; NSABP Foundation and NRG Oncology Biobank, Pittsburgh, PA, USA; NSABP Foundation and NRG Oncology, Pittsburgh, PA, USA; Department of Biostatistics, Graduate School of Public Health, Pittsburgh, PA, USA; NSABP Foundation and NRG Oncology, Pittsburgh, PA, USA; Orlando Health UF Health Cancer Center, Orlando, FL, USA; NSABP Foundation and NRG Oncology, Pittsburgh, PA, USA; UPMC Hillman Cancer Center, Magee Women’s Hospital University of Pittsburgh, Pittsburgh, PA, USA; NSABP Foundation and NRG Oncology, Pittsburgh, PA, USA; San Juan MBCCOP, San Juan, PR, USA; NSABP Foundation and NRG Oncology, Pittsburgh, PA, USA; Department of Medical Oncology, Cross Cancer Institute, Edmonton, Alberta, Canada; NSABP Foundation and NRG Oncology, Pittsburgh, PA, USA; Stroger Hospital Cook County MU NCORP, Chicago, IL, USA; NSABP Foundation and NRG Oncology, Pittsburgh, PA, USA; Centre Hospitalier de l’Université de Montréal, Montréal, QC, Canada; NSABP Foundation and NRG Oncology, Pittsburgh, PA, USA; NSABP Foundation and NRG Oncology Biobank, Pittsburgh, PA, USA; Syntax for Science SL, Palma de Mallorca, Spain; Inbiomotion SL, Barcelona, Spain; Inbiomotion SL, Barcelona, Spain; NSABP Foundation and NRG Oncology, Pittsburgh, PA, USA; Women’s Malignancies Disease Group, The Johns Hopkins Kimmel Comprehensive Cancer Center, Baltimore, MD, USA; NSABP Foundation and NRG Oncology, Pittsburgh, PA, USA; Kaiser Permanente Medical Center, Vallejo, CA, USA; NSABP Foundation and NRG Oncology, Pittsburgh, PA, USA; Department of Biostatistics, Graduate School of Public Health, Pittsburgh, PA, USA; Cancer Science, Institute for Research in Biomedicine (IRB Barcelona), The Barcelona Institute of Science and Technology, Barcelona, Spain; CIBERONC, Spain; ICREA, Institució Catalana de Recerca i Estudis Avançats, Barcelona, Spain; Universitat de Barcelona, Barcelona, Spain

## Abstract

**Background:**

The Adjuvant Zoledronic Acid (ZA) study in early breast cancer (AZURE) showed correlation between a nonamplified *MAF* gene in the primary tumor and benefit from adjuvant ZA. Adverse ZA outcomes occurred in MAF-amplified patients. NSABP B-34 is a validation study.

**Methods:**

A retrospective analysis of *MAF* gene status in NSABP B-34 was performed. Eligible patients were randomly assigned to standard adjuvant systemic treatment plus 3 years oral clodronate (1600 mg/daily) or placebo. Tumors were tested for *MAF* gene amplification and analyzed for their relationship to clodronate for disease-free survival (DFS) and overall survival (OS) in *MAF* nonamplified patients. All statistical tests were 2-sided .

**Results:**

*MAF* status was assessed in 2533 available primary tumor samples from 3311 patients. Of these, 37 withdrew consent; in 77 samples, no tumor was found; 536 assays did not meet quality standards, leaving 1883 (77.8%) evaluable for *MAF* assay by fluorescence in situ hybridization (947 from placebo and 936 from clodronate arms). At 5 years, in *MAF* nonamplified patients receiving clodronate, DFS improved by 30% (hazard ratio = 0.70, 95% confidence interval = 0.51 to 0.94; *P *=* *.02). OS improved at 5 years (hazard ratio = 0.59, 95% confidence interval = 0.37 to 0.93; *P *=* *.02) remaining statistically significant for clodronate throughout study follow-up. Conversely, adjuvant clodronate in women with *MAF*-amplified tumors was not associated with benefit but rather possible harm in some subgroups. Association between MAF status and menopausal status was not seen.

**Conclusions:**

Nonamplified *MAF* showed statistically significant benefits (DFS and OS) with oral clodronate, supporting validation of the AZURE study.

Post hoc subset analyses of the National Surgical Adjuvant Breast and Bowel Project (NSABP) B-34, AZURE, and other studies showed that benefits with adjuvant bisphosphonates (oral clodronate and IV zoledronic acid [ZA], respectively) for patients with early-stage breast cancer were limited to older or postmenopausal patients (1,2). This observation was supported by an individual patient meta-analysis of adjuvant bisphosphonate (BSP) trials by the Early Breast Cancer Trialists Collaborative Group (EBCTCG) (3). Statistically significant reductions in breast cancer–related mortality were observed in the 11 767 postmenopausal women receiving BSPs (hazard ratio [HR] = 0.82, 95% confidence interval [CI] = 0.73 to 0.93; *P *=* *.002), leading to European (4) and North American (5) clinical practice guidelines. However, acceptance has been slow. Imprecise definition of the benefiting patient subset is one possible reason.

Retrospective analysis of the Adjuvant Zoledronic Acid Study in Early Breast Cancer (AZURE) study showed correlation between a nonamplified *MAF* gene in the primary tumor biopsy and statistically significant beneficial effects of adjuvant ZA; adverse effects with ZA were seen in *MAF* -amplified patients (6). These results merited further investigation of *MAF* gene as a potential companion diagnostic in adjuvant BSP’s use in early-stage breast cancer. Validation is required before this biomarker can be routinely used according to recent ESMO bone health in cancer guidelines (7). NSABP B-34 is analyzed here as a validation study (8).

*MAF* amplification (at 16q23) leads to overexpression of MAF (mesenchymal aponeurotic fibrosarcoma gene, an AP-1 family transcription factor) in the primary tumor. This is associated with increased metastasis, especially bone metastasis (9). *MAF* transcriptionally controls genes, such as *CD36* and *PTHrP* (10,11), which regulate metastasis-related cellular processes, including survival, initiation, metabolic rewiring, and particularly, adhesion to bone marrow–derived cells and osteoclast differentiation (12). These observations point to *MAF* having a hierarchical role in metastasis.

*MAF* gene amplification was tested retrospectively in the AZURE trial of adjuvant ZA (1) and showed an association between overall survival (OS) benefit from ZA therapy and lack of an amplified *MAF* gene in the primary tumor (HR_OS_ =  0.69, 95% CI = 0.50 to 0.94; *P *=* *.02). In contrast, patients with *MAF*-amplified tumors who were non-postmenopausal (defined as >5 years postamenorrhea) at treatment start showed poor outcomes with ZA (HR_OS_ = 2.28, 95% CI = 1.07 to 4.82; *P *=* *.03). This difference between *MAF* groups resulted in a net zero effect in the AZURE trial. Using *MAF* as a biomarker showed that *MAF*-negative patients (both postmenopausal and previously unappreciated non-postmenopausal women) were likely to benefit from adjuvant ZA treatment (1,6). This category B analysis of the AZURE trial did not provide a high enough evidence level to propose changes in patient management.

NSABP B-34 is now analyzed as a validation study using archival tissues from this category B study similarly designed, conducted, and analyzed (13).

## Methods

### Study Design and Patients

NSABP B-34 was a randomized, placebo-controlled, double-blind trial of 3323 women with operable breast cancer (stages I-III) at 162 centers in Canada and the United States (NCT00009945). Estrogen (ER) and progesterone receptor (PgR) status was determined (HER2 status testing was not routine in North America at trial start in 2001). Before randomization, patients had a history and physical examination, blood work, and bone scans with radiographs (if indicated). Study procedures and approvals were in accordance with each center’s ethics committee guidelines and the Declaration of Helsinki. Patients gave pre-entry written informed consent.

### Randomization and Masking

At the NSABP Biostatistical Center (Pittsburgh, PA, USA), eligible patients were randomly assigned (1:1) postsurgery to adjuvant systemic treatment plus 3 years of either oral clodronate (1600 mg daily) or placebo. Participants were masked to group assignment. Stratified randomization was by biased-coin minimization. Patients were stratified (within each center) by age (younger than 50 or 50 years or older), number of positive axillary nodes (0, 1-3, or ≥4), and hormone receptor status. At relapse, study masking and drug was maintained if the patient was bone-metastasis free.

### Procedures

Patients received postsurgical local and systemic treatments. If indicated, chemotherapy was given concurrently with study drugs; endocrine therapy was administered for 5 years, with treatment choice at investigator’s discretion. Study drugs were discontinued if bone metastasis was detected. Patient follow-up included history and physical examination (with blood work) every 6 months for 5 years and annually thereafter with mammograms (2).

*MAF* gene amplification testing by a central laboratory (Targos Molecular Pathology, Kassel, Germany) was blinded and followed prespecified sample handling and standard operating methodology. Scoring was the same as for the previous AZURE analyses (6) but using full-tissue sections (5-µm thick). Slices were first analyzed for evaluable tumor using hematoxylin and eosin staining. *MAF* amplification was assessed using the analytically validated (*MAF*/D16Z3) fluorescence in situ hybridization (FISH) test MAFTEST (Inbiomotion, Barcelona, Spain).

Mean *MAF* copy number per nucleus was established from 50 nuclei in tumor regions with highest amplification. Sections were assessed by FISH once, with no option for optimization. A single repetition was allowed if FISH failure was tissue related (eg, section too thick or tissue washed off). Patients were scored as *MAF* positive, indicating *MAF* amplified, with a mean number of 2.5 or more *MAF* copies per nucleus (as defined for the AZURE trial). *MAF* negative means *MAF* nonamplified.

### Statistical Analysis

The primary objective was to test the predictive association between *MAF* status and adjuvant clodronate outcomes. Endpoint analyses included all patients with follow-up information who were scored in the *MAF* assay. Women withdrawing consent after randomization were excluded. The statistical analysis plan was prespecified and agreed between Division of Biostatistics and Science NRG Oncology (USA), the NSABP, and Syntax For Science (Spain).

The primary endpoint was disease-free survival (DFS) in *MAF*-negative patients, defined as time from randomization to local, regional, or distant recurrence, contralateral breast cancer, second primary cancer, or death from any cause before breast cancer recurrence. Secondary endpoints were OS (time from randomization to death from any cause), recurrence-free interval (RFI; time from randomization to local, regional, or distant breast cancer recurrence, not including contralateral breast cancer and death from breast cancer), bone metastasis-free interval, and nonbone metastasis-free interval. A hierarchical approach for reduction of alpha consumption was considered for DFS and OS in *MAF*-nonamplified patients.

Predictive analyses of *MAF* status with treatment allocation used multivariate modeling (Cox proportional hazards model) to adjust for age (2-level factor fitted: 49 years or younger compared with 50 years or older); nodal status (3-level factor: negative, 1-3, ≥4); presence of ER and PgR (2-level factor: both negative, positive for at least 1); histological grade (4-level factor: low, intermediate, high, or missing); and pathological tumor size (4-level factor: ≤2.0 cm, 2.1-4 cm, ≥4.1 cm, unknown). Analyses (including trend analyses) were done at years 5 and 7 and at complete follow-up, given the heterogeneity of treatment regimens between clodronate and ZA (2,14). Menopause and age were included as exploratory endpoints, as no statistically significant heterogeneity of treatment effects within the subgroup of *MAF* negative was observed in AZURE (1,6). The interaction of *MAF* amplification (positive or negative) with treatment allocation has been performed as described before (15).

Prognostic values of *MAF* status for DFS and OS of patients with early-stage breast cancer in the placebo control group only were investigated as exploratory objectives using survival curves, whereas the endpoints of time to bone metastasis or nonbone metastasis were assessed using cumulative incidence function curves, with death as a competing event. Median survival and incidence rate were estimated using the Kaplan-Meier method. Hazard ratios were obtained using Cox proportional hazards models. The 95% confidence interval values are based on profile-likelihood method, and *P* values are based on likelihood ratio. Hypothesis testing was 2-sided at 5% statistical significance level. All analyses were carried out via SAS, version 9.4 (SAS Institute, Cary, NC).

Statistical analyses were done at the Syntax For Science (Palma de Mallorca, Spain) and revised and reviewed by the Division of Biostatistics and Science NRG Oncology (Pittsburgh, PA, USA).

## Results

**Figure 1.**
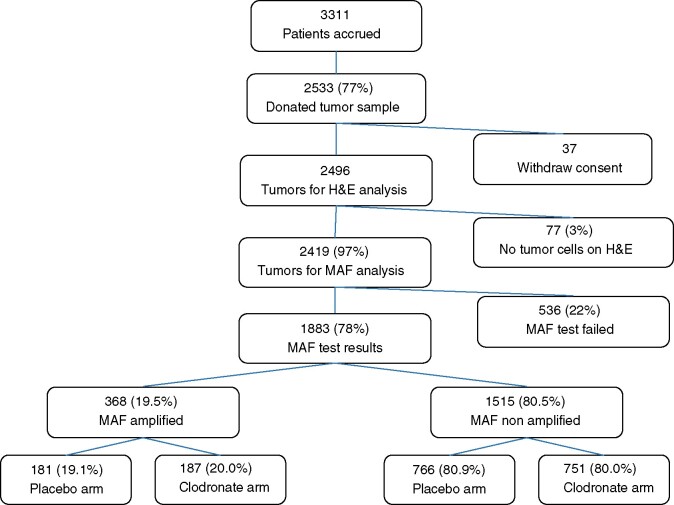
Schematic representation depicting *MAF* test analyses on the NSABP B-34 patient population. The *MAF* status distribution according to patient subgroups is reported. H&E = hematoxilyin and eosin.

We analyzed the *MAF* status of 2533 (76.5%) tumor samples from 3311 patients recruited to the B-34 study between January 22, 2001, and March 31, 2004 (Figure 1); 37 patients were ineligible because of consent withdrawal, giving a total of 2496 samples. An invasive tumor was hematoxylin and eosin confirmed in 96.9% (2419 patients), and the *MAF* FISH assay was performed on these samples on adjacent sections and assessed using the stringent quality standards of TARGOS Molecular Pathology. Of these 2419 patients, 1883 (77.8%) were evaluable by *MAF* assay providing FISH results. *MAF*-amplified (*MAF*-positive) tumors were found for 368 (19.5%) of these patients, similar to the 21% reported in the AZURE cohort (6). Overall, *MAF*-evaluable patients (primary analysis set, n = 1883) represented 56.9% of the total B-34 patient cohort, with 947 in the placebo arm and 936 in the clodronate arm.

Median follow-up was 109.5 months (interquartile range = 97.5-120). In this biomarker subset, 406 patients had a DFS event (206 placebo, 200 clodronate), 241 an OS event (133 placebo, 108 clodronate), 236 an RFI event (123 placebo, 113 clodronate), and 88 a bone skeletal event (51 placebo, 37 clodronate). Patient characteristics (Table 1) and rates of DFS and OS events for the clodronate and placebo arms of the subgroup of *MAF* evaluable patients were similar to the overall B-34 study (Supplementary Table 1, available online). Approximately two-thirds of women were 50 years or older on entry, and three-quarters had negative axillary nodes and ER positivity. Both chemotherapy and hormone treatment (mostly tamoxifen) were administered to 45.5% (426 of 936) and 45.6% (432 of 947) of patients receiving clodronate and placebo, respectively (Table 1). Of the patients, 21.6% (clodronate) and 20.4% (placebo) received chemotherapy alone and 27.4% and 27.5% of the clodronate and placebo received hormone therapy alone.

**Table 1.** Patient clinical characteristics in all B-34 patients according to treatment arm and *MAF* amplification status

	Paterson et al. (2)^a^		*MAF* evaluable samples^b^			
Clinical parameter and subgroups	Placebo (n = 1656)^c^	Clodronate (n = 1655)^c^	Placebo (n = 947)	Clodronate (n = 936)	*MAF* negative (n = 1515)	*MAF* positive (n = 368)
Median (IQR) follow-up, mo^d^	91.5 (83.4-100.0)	90.0 (82.3-100.0)	111.4 (103.4- 122.1)	111.3 (101.7-121.3)	111.3 (102.7-121.6)	111.8 (101.5-122.2)
Age at entry, No. (%)						
≤49 y	589 (35.5)	594 (35.7)	332 (35.1)	336 (35.9)	526 (34.7)	142 (38.6)
≥50 y	1072 (64.5)	1068 (64.3)	615 (64.9)	600 (64.1)	989 (65.3)	226 (61.4)
Ethnic origin, No. (%)						
White	1375 (82.8)	1381 (83.1)	801 (84.6)	784 (83.8)	1294 (85.4)	291 (79.1)
Hispanic	90 (5.4)	96 (5.8)	41 (4.3)	49 (5.2)	71 (4.7)	19 (5.2)
Black	126 (7.6)	117 (7.0)	63 (6.7)	71 (7.6)	89 (5.9)	45 (12.2)
Pacific Islander	9 (<1)	4 (<1)	7 (0.7)	1 (0.1)	6 (0.4)	2 (0.5)
Asian	43 (2.6)	48 (2.9)	21 (2.2)	23 (2.5)	37 (2.4)	7 (1.9)
American Indian	3 (<1)	6 (<1)	3 (0.3)	3 (0.3)	6 (0.4)	0 (0.0)
Other	10 (<1)	8 (<1)	7 (0.7)	3 (0.3)	6 (0.4)	4 (1.1)
Unknown	5 (<1)	2 (<1)	4 (0.4)	2 (0.2)	6 (0.4)	0 (0.0)
No. of positive nodes, No. (%)						
Negative	1252 (75.4)	1258 (75.7)	737 (77.8)	694 (74.1)	1159 (76.5)	272 (73.9)
1-3	295 (17.8)	296 (17.8)	147 (15.5)	176 (18.8)	259 (17.1)	64 (17.4)
4 or more	114 (6.9)	108 (6.5)	63 (6.7)	66 (7.1)	97 (6.4)	32 (8.7)
Hormone receptor status, No. (%)						
Both negative	368 (22.2)	368 (22.1)	202 (21.3)	223 (23.8)	247 (16.3)	178 (48.4)
Either or both positive	1293 (77.8)	1294 (77.9)	745 (78.7)	713 (76.2)	1268 (83.7)	190 (51.6)
Adjuvant therapy, No. (%)						
Chemotherapy alone	344 (20.8)	342 (20.7)	193 (20.4)	203 (21.6)	238 (15.7)	158 (42.9)
Hormonal therapy alone	518 (31.3)	512 (30.9)	260 (27.5)	257 (27.4)	470 (31.0)	47 (12.8)
Both	720 (43.5)	728 (44.0)	432 (45.6)	426 (45.5)	713 (47.1)	145 (39.4)
None	53 (3.2)	51 (3.1)	28 (3.0)	26 (2.8)	39 (2.6)	15 (4.1)
Unknown	26 (1.6)	29 (1.8)	8 (0.8)	9 (1.0)	13 (0.9)	4 (1.1)
Pathological tumor size, No. (%)						
≤2.0 cm	1119 (67.4)	1127 (67.8)	640 (67.6)	617 (65.9)	1045 (69.0)	212 (57.6)
2.1-4.0 cm	456 (27.5)	466 (28.0)	261 (27.6)	282 (30.1)	410 (27.1)	133 (36.1)
≥4.1 cm	81 (4.9)	64 (3.9)	46 (4.9)	37 (4.0)	60 (4.0)	23 (6.3)
Unknown	5 (<1)	5 (<1)				
Histological grade, No. (%)						
Low	374 (22.5)	377 (22.7)	198 (20.9)	196 (20.9)	370 (24.4)	24 (6.5)
Intermediate	665 (40.0)	667 (40.1)	386 (40.8)	378 (40.4)	661 (43.6)	103 (28.0)
High	589 (35.5)	575 (34.6)	351 (37.1)	344 (36.8)	456 (30.1)	239 (64.9)
Unknown	28 (1.7)	36 (2.2)	12 (1.3)	18 (1.9)	28 (1.8)	2 (0.5)

a 3323 patients were randomly assigned: 1661 to placebo and 1662 to clodronate. There were 3311 patients with follow-up data. IQR = interquartile range.

b There were 2533 tumor samples for *MAF* test and 1883 *MAF* evaluable patients in the primary set.

c Patients with follow-up data.

d Based on 1648 patients reported to be alive at last follow-up.

The frequency of *MAF*-positive status was unaffected by age or axillary lymph node status but was more common with poor histological grade, ER negativity, and larger tumors (Table 1). Consequently, women with *MAF*-positive tumors were more likely to have received chemotherapy and less likely to have had endocrine treatments than the group as a whole. Given the heterogeneity of the prognostic factors in the baseline demographics, predictive results are reported using multivariate Cox modeling, adjusted by age, nodal status, ER and PgR status, histological grade, and pathological tumor size.

**Figure 2.**
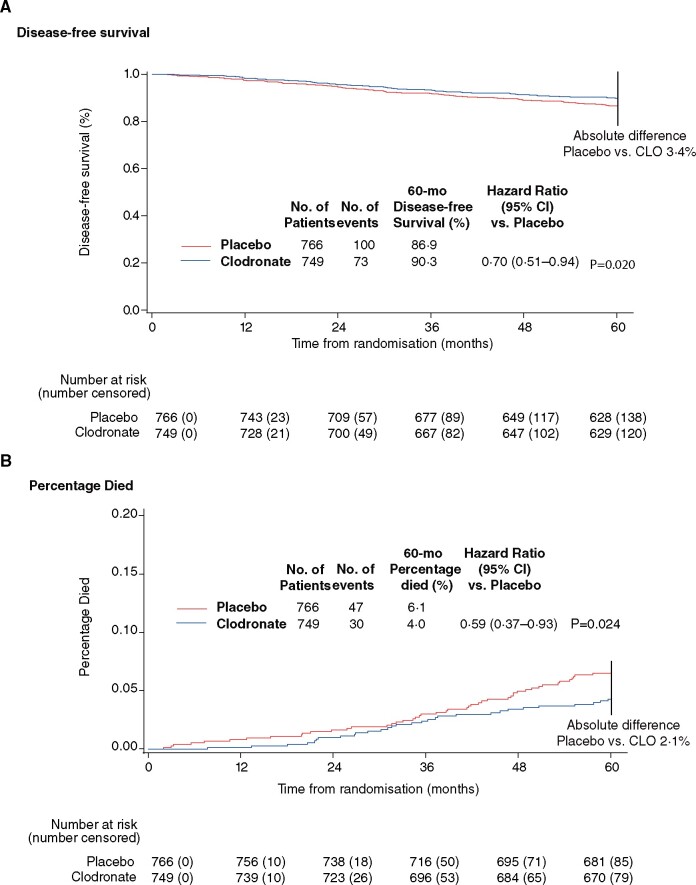
Disease-free survival (**A**) and percentage who died (**B**) by treatment group in *MAF*-nonamplified patients at 5-year follow-up from Cox multivariable model adjusted for differences in age, nodal status, presence of estrogen and progesterone, histological grade, and pathological tumor size. All statistical tests are 2-sided. CI = confidence interval; CLO = Clodronate; HR = hazard ratio .

For patients with *MAF*-nonamplified tumors (*MAF* negative), clodronate treatment showed improved DFS at 5 years compared with placebo (HR = 0.70, 95% CI = 0.51 to 0.94; *P *=* *.02) (Figure 2, A), although this association is no longer statistically significant with longer follow-up times (Table 2;Supplementary Figure 1, A, available online). At 5 years, patients with *MAF*-negative tumors had a 41.0% reduction in hazard for death (HR = 0.59, 95% CI = 0.37 to 0.93; *P *=* *.02) (Figure 2, B). Fewer patients with *MAF*-negative tumors treated with clodronate died (n = 30; 4.0%) compared with the placebo group (n = 47; 6.1%). Next, we tested *MAF* and treatment interaction even though the *MAF*-negative subgroup and its *MAF*-positive complement are different in size (4 to 1) and the heterogeneity test loses power. Yet, under these unfavorable circumstances and although the study was not powered to detect treatment by *MAF* status interactions, a clear trend is detected comparing the OS outcomes of *MAF* (+) vs (–) patients with a *P*_interaction_ of .06. The benefit in OS in *MAF*-negative patients was statistically significant at all timepoints (Table 2;Supplementary Figure 1, B, available online); these benefits were not observed for *MAF*-positive patients (Table 2) or for the overall cohort (Supplementary Table 1, available online) and did not associate statistically significantly with age or menopausal status (younger than 50 years: HR = 0.80, 95% CI = 0.43 to 1.49; and 50 years or older: HR = 0.72, 95% CI = 0.51 to 1.02) (Table 3). The clodronate group’s absolute death risk reduction at complete follow-up in *MAF*-negative patients was 2.8% (from 12.4% for placebo to 9.6% for clodronate, a 22.6% reduction in death).

**Table 2.** Outcomes of the trial endpoints according to patients subgroups and follow-up time^a^

Endpoint	5 years		7 years		Median follow of 109 months	
	HR (95% CI)	*P*	HR (95% CI)	*P*	HR (95% CI)	*P*
*MAF* negative (n = 1515)						
OS	0.59 (0.37 to 0.93)	.02	0.69 (0.48 to 1.00)	.047	0.74 (0.54 to 1.00)	.046
DFS	0.70 (0.51 to 0.94)	.02	0.83 (0.64 to 1.07)	.15	0.93 (0.74 to 1.16)	.52
RFI	0.67 (0.45 to 0.98)	.04	0.82 (0.58 to 1.14)	.24	0.91 (0.67 to 1.23)	.53
Bone metastases	0.89 (0.47 to 1.68)	.71	0.79 (0.46 to 1.37)	.40	0.82 (0.50 to 1.34)	.43
Nonbone metastases	0.70 (0.42 to 1.17)	.17	0.82 (0.52 to 1.28)	.38	0.73 (0.48 to 1.10)	.13
*MAF* positive (n = 368)						
OS	1.39 (0.71 to 2.70)	.34	1.26 (0.70 to 2.28)	.44	1.01 (0.62 to 1.67)	.96
DFS	1.00 (0.62 to 1.61)	1.00	1.05 (0.67 to 1.64)	.83	1.06 (0.70 to 1.59)	.79
RFI	0.83 (0.46 to 1.49)	.53	0.78 (0.45 to 1.35)	.37	0.81 (0.49 to 1.35)	.42
Bone metastases	0.44 (0.14 to 1.39)	.16	0.33 (0.11 to 0.98)	.046	0.40 (0.15 to 1.06)	.06
Nonbone metastases	0.70 (0.31 to 1.55)	.38	0.60 (0.28 to 1.26)	.17	0.62 (0.30 to 1.27)	.19

a All statistical tests were 2-sided. CI = confidence interval; DFS = disease-free survival; HR = hazard ratio; OS = overall survival; RFI = recurrence-free interval.

**Table 3.** Outcomes of the trial endpoints according to patients subgroups^a^

Endpoint	≤49 y		≥50 y		Premenopausal		Postmenopausal	
	*MAF* negative	*MAF* positive	*MAF* negative	*MAF* positive	*MAF* negative	*MAF* positive	*MAF* negative	*MAF* positive
	(n = 526)	(n = 142)	(n = 989)	(n = 226)	(n = 466)	(n = 111)	(n = 535)	(n = 138)
	HR (95% CI)	HR (95% CI)	HR (95% CI)	HR (95% CI)	HR (95% CI)	HR (95% CI)	HR (95% CI)	HR (95% CI)
DFS	0.98 (0.63 to 1.51)	0.73 (0.36 to 1.43)	0.91 (0.70 to 1.18)	1.23 (0.75 to 2.04)	1.14 (0.68 to 1.90)	0.88 (0.43 to 1.79)	0.83 (0.59 to 1.16)	1.42 (0.75 to 2.79)
OS	0.80 (0.43 to 1.49)	0.91 (0.37 to 2.17)	0.72 (0.51 to 1.02)	0.96 (0.54 to 1.71)	0.88 (0.41 to 1.86)	1.02 (0.38 to 2.68)	0.72 (0.46 to 1.12)	1.20 (0.58 to 2.53)
RFI	1.06 (0.64 to 1.76)	0.60 (0.26 to 1.31)	0.85 (0.58 to 1.23)	0.96 (0.50 to 1.84)	1.25 (0.68 to 2.33)	0.85 (0.38 to 1.84)	0.74 (0.45 to 1.22)	1.05 (0.44 to 2.52)
Bone metastases	1.32 (0.60 to 3.03)	0.34 (0.05 to 1.37)	0.61 (0.32 to 1.15)	0.42 (0.11 to 1.35)	1.78 (0.62 to 5.78)	0.49 (0.11 to 1.78)	0.59 (0.26 to 1.29)	0.45 (0.06 to 2.31)
Nonbone metastases	0.77 (0.38 to 1.51)	0.64 (0.17 to 2.06)	0.74 (0.44 to 1.23)	0.57 (0.23 to 1.32)	0.76 (0.33 to 1.72)	0.70 (0.18 to 2.34)	0.69 (0.35 to 1.32)	0.64 (0.21 to 1.84)

a CI = confidence interval; DFS = disease-free survival; HR = hazard ratio; OS = overall survival; RFI = recurrence-free interval.

RFI showed an association between treatment and *MAF* status at 5-year follow-up similar to DFS. Statistically significantly fewer patients with *MAF*-negative tumors in the clodronate patients had breast cancer–related events (n = 45; 6%) than those in the placebo group (n = 60, 7.8%; HR = 0.67, 95% CI = 0.45 to 0.98; *P = *.04) (Table 2). For *MAF*-positive patients, no clodronate treatment effect was observed for DFS, RFI, and OS at different follow-up times (Table 2), with a nonsignificant association to poorer OS—more evident in times closer to the treatment period. In exploratory analyses, we tested the impact of clodronate on bone and nonbone metastasis in *MAF*-positive and *MAF*-negative subgroups, but a statistically significant association was absent likely because of low event numbers.

To determine that *MAF* negativity or positivity associates with DFS and OS (ie, is prognostic), we assessed its influence on outcome in the placebo control group. Disease progression was observed for 48 of 181 (26.5%) patients with *MAF*-positive tumors and for 158 of 766 (20.6%) patients with *MAF*-negative tumors (Supplementary Table 2, available online) confirming that *MAF* status was prognostic (HR = 1.39, 95% CI = 1.01 to 1.92; *P *=* *.045) for DFS (Supplementary Figure 2, A, available online). *MAF* status was also prognostic for OS, RFI, and metastasis (bone and nonbone) (Table 4; Supplementary Figure 2, B, available online).

**Table 4.** Prognostic of outcome in placebo control arm patients (n = 947)^a^

Endpoint and *MAF* group	No.^b^	Univariable HR (95% CI)	*P* ^c^
DFS	947	1.39 (1.01 to 1.92)	.045
OS	947	1.59 (1.59 to 2.33)	.02
RFI	947	1.88 (1.27 to 2.77)	.02
Bone metastases	947	2.03 (1.13 to 3.68)	.02
Nonbone metastases	947	1.81 (1.09 to 3.00)	.02

a Median follow up. CI = confidence interval; DFS = disease-free survival; HR = hazard ratio (MAF+ve/-ve); OS = overall survival; RFI = recurrence-free interval.

b 766 *MAF*-negative patients and 181 *MAF*-positive patients.

c All statistical tests are 2-sided.

## Discussion

Bisphosphonates are bone-targeting drugs binding to sites of bone resorption; after internalization by osteoclasts, bisphosphonates inhibit their function. Although rapidly cleared from the circulation, they have a long bone half-life and are effective in conditions with excessive bone resorption, including osteoporosis. They are useful in managing bone metastases in breast cancer–reducing skeletal–related events and improving life quality (7,16).

The bisphosphonates clodronate and ZA differ in how they inhibit osteoclast activity and induce apoptosis: amino-bisphosphonates (such as ZA) inhibit farnesyl pyrophosphate synthase (an enzyme crucial to cell growth and division), whereas nonamino-bisphosphonates (eg, clodronate) form cytotoxic metabolites (17). The SWOG S0307 (18) study confirmed that clodronate and ZA give equivalent outcomes in adjuvant breast cancer.

In an early open-label study, clodronate reduced bone metastasis frequency and increased survival in women with evidence of disseminated bone marrow cancer cells (19), but 2 subsequent trials gave conflicting results. A large placebo-controlled study by Powles et al. (20) showed statistically significant benefit for bone metastasis-free survival and overall survival in all women with breast cancer receiving adjuvant oral clodronate. However, a smaller open-label study (mainly in premenopausal women) suggested possible potential harm from clodronate (21). In 2012, Gnant et al. (22) reported a survival benefit with ZA for women receiving ovarian suppression plus tamoxifen or anastrozole for ER-positive cancer.

Looking for confirmatory studies, 2 larger trials over longer periods of adjuvant bisphosphonate treatment (clodronate for 3 years and ZA for 5 years) commenced (2,14). Neither trial met its primary endpoint (DFS) although retrospective subset analyses in B-34 showed that patients older than 50 years or who were postmenopausal more than 5 years in AZURE had statistically significant treatment benefits with adjuvant clodronate and ZA. This suggested that adjuvant bisphosphonates might be efficacious for postmenopausal patients. Subsequently, an individual patient meta-analysis by the EBCTCG (3) showed a clear reduction in breast cancer mortality in postmenopausal patients (HR_OS_ = 0.82, 95% CI = 0.73 to 0.93; 2-sided *P *=* *.002). This influential meta-analysis led to guidelines recommending adjuvant IV ZA or oral clodronate for postmenopausal women to reduce relapse in bone and overall survival (4,5). Because of low event numbers for bone metastases (51 placebo vs 37 clodronate), we are unable to comment specifically on bone relapse. However, the lack of a clear mechanistic explanation why bisphosphonates benefit only a subgroup of often hard-to-define patients is an unmet medical need defined in ASCO (5) and ESMO bone health in cancer guidelines (7). The latter states that the *MAF* biomarker may predict benefit (and harm) from bone microenvironment manipulation. Validation in an independent trial is required before routine use (7). Clodronate and ZA both act on osseous stroma and not on tumor cells and are considered clinically equi-efficacious. Difference in pharmacological potency may account for the harm seen in *MAF*-positive patients in AZURE, though this is conjecture at this point.

The hypothesis that a negative *MAF* gene amplification result (*MAF* negative) measured by FISH might predict benefit from adjuvant clodronate treatment came from the AZURE study of ZA (1,6). In the B-34 study, we tested tissue samples, paraffin-fixed and archived for 15-19 years, using current standard practices. Of the samples, 3% had insufficient tumor tissue, and 25% were unevaluable by the *MAF* assay because of long storage times, explaining the attrition of patients for MAFTEST testing; however, this still met criteria for the preplanned statistical analyses. Of note, in samples stored less than 1 year, the rate of FISH failure was lower (2%-5%) and mostly attributable to the lack of relevant tumor tissue for analysis. The available two-thirds of the total accrued patients’ specimens were representative of the original cohort. Adjuvant clodronate improved disease outcome (DFS and OS) for the 80.3% of patients with *MAF*-negative tumors independent of age or menopause at study entry. Conversely, the use of adjuvant clodronate in women with *MAF*-positive tumors was not associated with any treatment benefit, supporting the previously published data on the *MAF* biomarker in AZURE (1,6).

For a biomarker, interpretable evaluation using archived tissues requires the assay to reflect what would happen in a clinical setting. The current analysis provides level-1 evidence by presenting a category B confirmation of a previous category B hypothesis-generating study (13). In AZURE, *MAF*-negative patients receiving ZA had a statistically significantly improved invasive DFS (IDFS ) and survival, whereas *MAF*-positive patients had a reduced IDFS and survival. In NSABP B-34, we confirm that *MAF*-negative patients have a statistically significant improvement in DFS and OS, whereas *MAF*-positive patients had no benefit with adjuvant bisphosphonates. The absolute death risk reductions at 5 years were 5.8% and 2.8%, respectively (a 25.3% reduction in DFS and 35.7% reduction in death events on clodronate treatment). These are in line with those for IDFS for pertuzumab in adjuvant treatment of HER2-positive breast cancer patients (23) and of higher magnitude than those reported by NICE (24) to approve the use of bisphosphonates in postmenopausal patients in the United Kingdom on the basis of the EBCTCG meta-analysis (3). The confirmation in a study with a high proportion of stage I patients as opposed to stage II and III disease in AZURE has implications for routine clinical practice. At present, the guidelines suggest treatment is limited to patients at intermediate to high risk of relapse, perhaps reflecting some uncertainty over the best way to apply the EBCTCG findings to routine clinical practice. Clinical guidelines in Europe (4) and North America (5) might now be supported by an objective diagnostic test.

*MAF* biomarker selection should allow (with more precise identification of a benefiting subgroup) higher treatment adoption rates, including younger premenopausal patients currently excluded in guidelines. Our study shows (Table 1) 670 patients who were *MAF* evaluable were aged 49 years or younger (and likely clinically classified as premenopausal) with 526 being *MAF* negative (78.5%) who might have benefited from bisphosphonates. Data from these 2 studies suggest that beneficial effects of bisphosphonates for breast cancer are associated with the absence of an amplified *MAF* gene in the primary tumor (*MAF* negative); bisphosphonates do not improve outcome (and may harm) women with *MAF*-amplified tumors (*MAF* positive).

**Figure 3.**
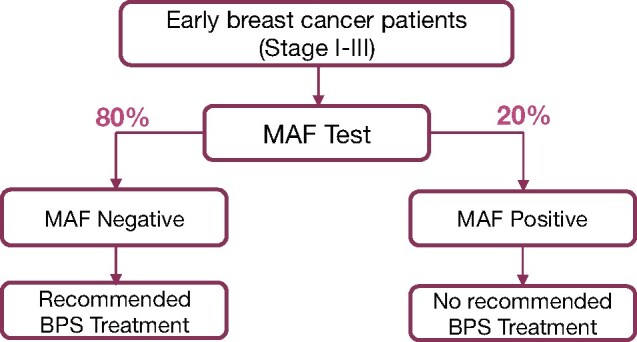
Schematic representation depicting adjuvant bisphosphonates recommended treatment algorithm based on MAF test .

According to *MAF*, as opposed to menopause (as currently recommended by guidelines), identifying *MAF*-negative patients could give a larger patient population the opportunity to benefit from adjuvant bisphosphonates while avoiding potential harm (or no benefit) than solely using menopausal status as a selection criterion (Figure 3).

## Funding

This work was supported by the US National Cancer Institute, National Surgical Adjuvant Breast and Bowel Project, Inbiomotion and Bayer Oy (formerly Schering Oy), and grants from the U10CA180868 (NCI Cancer Trial Network), UG1CA189867 (NCI Community Oncology Research Program), U10CA180822 (NRG Oncology SDMC [Biostats]), U24CA196067-04 (BSB) (Lab).

## Footnotes

**Role of the funders:** The funders did not have a role in the design of the study; the collection, analysis, and interpretation of the data; the writing of the manuscript; or the decision to submit the manuscript for publication. 

**Disclosures:** AB is consultant for Amgen, Novartis, Agendia. JJM owns less than 0.25% and JCT is an employee of Inbiomotion. RRG and JCT have patents pending related to this work. RRG declares shares of Inbiomotion (<US$25 000). The other authors declare no competing interests.

**Author contributions:** AHGP: Principal Investigator, Conceptualization, Author. PCL: Investigator, Resources. SJA: Statistician, Resources, Editing. EPM: Resources, Supervision, Editing. AB: Investigator, Resources, Review. LB-D: Investigator, Resources, Review. KMK: Investigator, Resources, Review. TL: Investigator, Resources, Review. AR: Investigator, Resources, Review. JW: Investigator, Resources, Review. MF: Resources, project administration. MS: Statistician, Review JCT: Investigator, Data Curation, Editing. JJM: Funding acquisition, Conceptualization, Editing. ACW: Investigator, Resources, Review. LF: Investigator, Resources, Review. NW: Investigator, Resources, Review. RRG: Conceptualization, Investigator, Methodology, Author.

**Acknowledgements:** We thank members of the NSABP group, and all women participating in NSABP B-34. The original trial was supported by the National Cancer Institute’s Department of Health and Human Services public health service grants with additional funding from Bayer Oy (formerly Schering Oy).

**Disclaimer:** The US Department of Health and Human Services specifically disclaims responsibility for any analysis, interpretations, or conclusions; and Schering-Plough Inc. 

## Data Availability 

All data published in this article and any related data will be available on request to the corresponding author.

## Supplementary Material

pkab054_Supplementary_Data
