# Supplementary material for: MAF Amplification and Adjuvant Clodronate Outcomes in Early-Stage Breast Cancer in NSABP B-34 and Potential Impact on Clinical Practice
Source: JNCI Cancer Spectr. 2021 May 28;5(4):pkab054. doi: 10.1093/jncics/pkab054 (PMC8346694; doi:10.1093/jncics/pkab054)
Supplement: pkab054_Supplementary_Data [file pkab054_supplementary_data.pdf]

SUPPLEMENTARY MATERIALS

Supplementary Tables

Supplementary Table 1. Outcomes HR of the trial endpoints according to patients' subgroups and follow-up time

	Endpoints	5 years	7 years	median FU=109m
All B-34 N=3311	OS	HR= 0.81 (0.61, 1.09) p= 0.15	HR= 0.86 (0.68, 1.10) p= 0.22	HR= 0.90 (0.74, 1.10) p= 0.29
	DFS	HR= 0.85 (0.70, 1.03) p= 0.10	HR= 0.92 (0.78, 1.09) p= 0.34	HR= 0.97 (0.83, 1.12) p= 0.65
	RFI	HR= 0.81 (0.64, 1.02) p= 0.07	HR= 0.89 (0.72, 1.09) p= 0.26	HR= 0.92 (0.76, 1.11) p= 0.38
	Bone mets	HR= 0.83 (0.56, 1.24) p= 0.36	HR= 0.77 (0.54, 1.10) p= 0.15	HR= 0.82 (0.59, 1.12) p= 0.21
	Non_Bone mets	HR= 0.67 (0.48, 0.93) p= 0.01	HR= 0.74 (0.55, 1.00) p= 0.04	HR= 0.72 (0.55, 0.96) p= 0.02
MAF Samples N=2419	OS	HR= 0.74 (0.53, 1.04) p= 0.08	HR= 0.83 (0.63, 1.09) p= 0.17	HR= 0.83 (0.66, 1.04) p= 0.10
	DFS	HR= 0.81 (0.65, 1.02) p= 0.07	HR= 0.89 (0.73, 1.08) p= 0.24	HR= 0.96 (0.81, 1.14) p= 0.63
	RFI	HR= 0.79 (0.59, 1.04) p= 0.09	HR= 0.89 (0.69, 1.14) p= 0.34	HR= 0.92 (0.73, 1.15) p= 0.45
	Bone Mets	HR= 0.85 (0.54, 1.36) p= 0.49	HR= 0.78 (0.52, 1.18) p= 0.24	HR= 0.79 (0.55, 1.15) p= 0.22
	Non_Bone mets	HR= 0.73 (0.50, 1.08) p= 0.11	HR= 0.82 (0.58, 1.15) p= 0.25	HR= 0.80 (0.58, 1.10) p= 0.16

Supplementary Table 2. Endpoint events in patient subgroups

	FULL B-34 (n= 3311)			MAF evaluable (n=1883)			MAF Negative (n=1515)			MAF Positive (n=368)		
	Placebo (n=1656)	Clodronate (n=1655)	Total	Placebo (n=947)	Clodronate (n=936)	Total	Placebo (n=766)	Clodronate (n=749)	Total	Placebo (n=181)	Clodronate (n=187)	Total
Events	N (%)	N (%)	N (%)	N (%)	N (%)	N (%)	N (%)	N (%)	N (%)	N (%)	N (%)	N (%)
DFS	355 (21.4)	347 (21.0)	702 (21.2)	206 (21.8)	200 (21.4)	406 (21.6)	158 (20.6)	150 (20.0)	308 (20.3)	48 (26.5)	50 (26.7)	98 (6.5)
OS	204 (12.3)	184 (11.1)	388 (11.7)	133 (14.0)	108 (11.5)	241 (12.8)	98 (12.8)	75 (10.0)	173 (11.4)	35 (19.3)	33 (17.6)	68 (4.5)
RFI	221 (13.3)	206 (12.4)	427 (12.9)	123 (13.0)	113 (12.1)	236 (12.5)	74 (9.7)	67 (8.9)	141 (9.3)	36 (19.9)	28 (15.0)	64 (4.2)
Bone Mets	85 (5.1)	70 (4.2)	155 (4.7)	51 (5.4)	37 (4.0)	88 (4.7)	34 (4.4)	31 (4.1)	65 (4.3)	16 (8.8)	6 (3.2)	22 (1.5)
Non-Bone Mets	115 (6.9)	84 (5.1)	199 (6.0)	73 (7.7)	55 (5.9)	128 (6.8)	52 (6.8)	42 (5.6)	94 (6.2)	21 (11.6)	13 (7.0)	34 (2.2)

Supplementary Figures

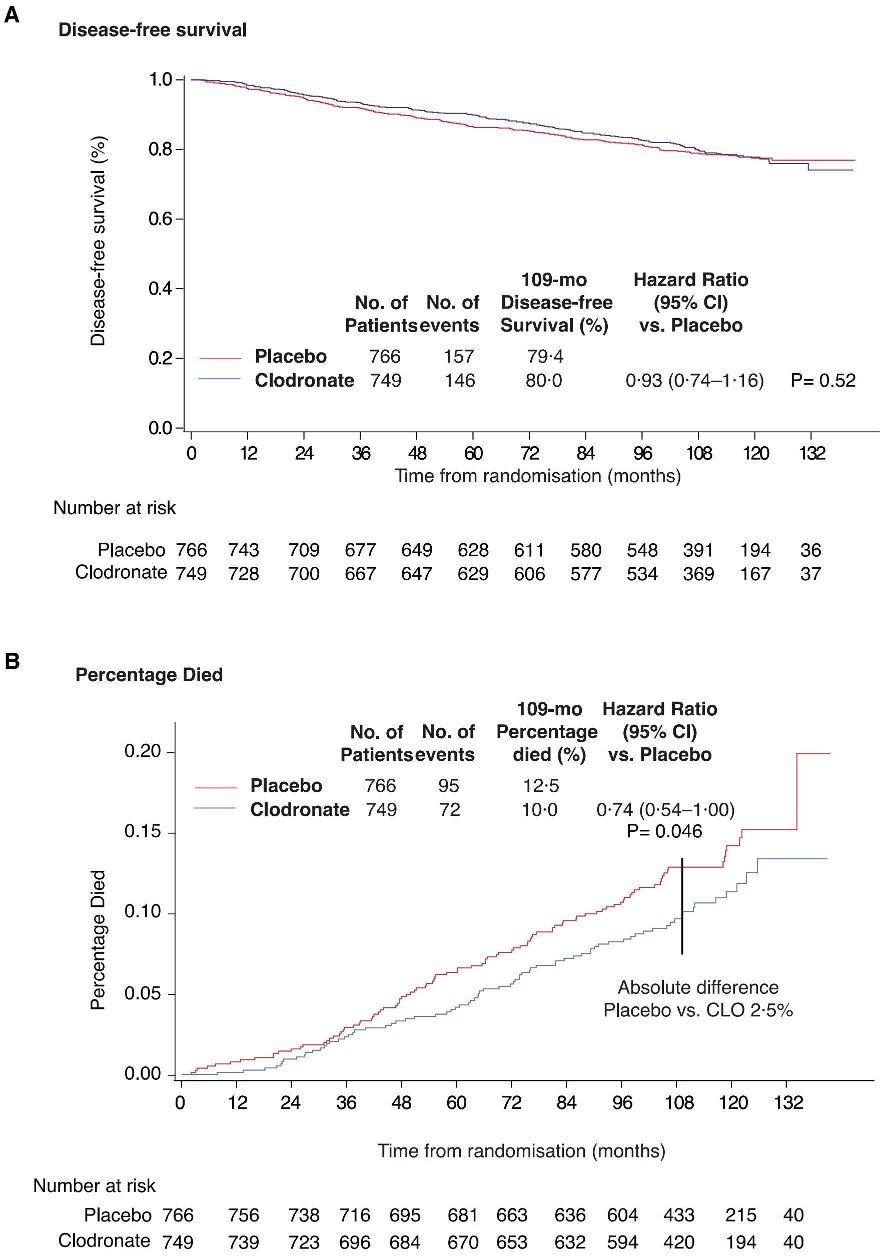

Supplementary Figure 1. Adjuvant clodronate outcomes according to MAF stratification A) Disease-free survival and B) Percentage died by treatment group in *MAF*-non-amplified patients at complete follow-up from Cox multivariable model adjusted for differences in age; nodal status; presence of ER/PgR; histological grade; and pathological tumor size. HR=hazard ratio, CI=Confidence Interval.

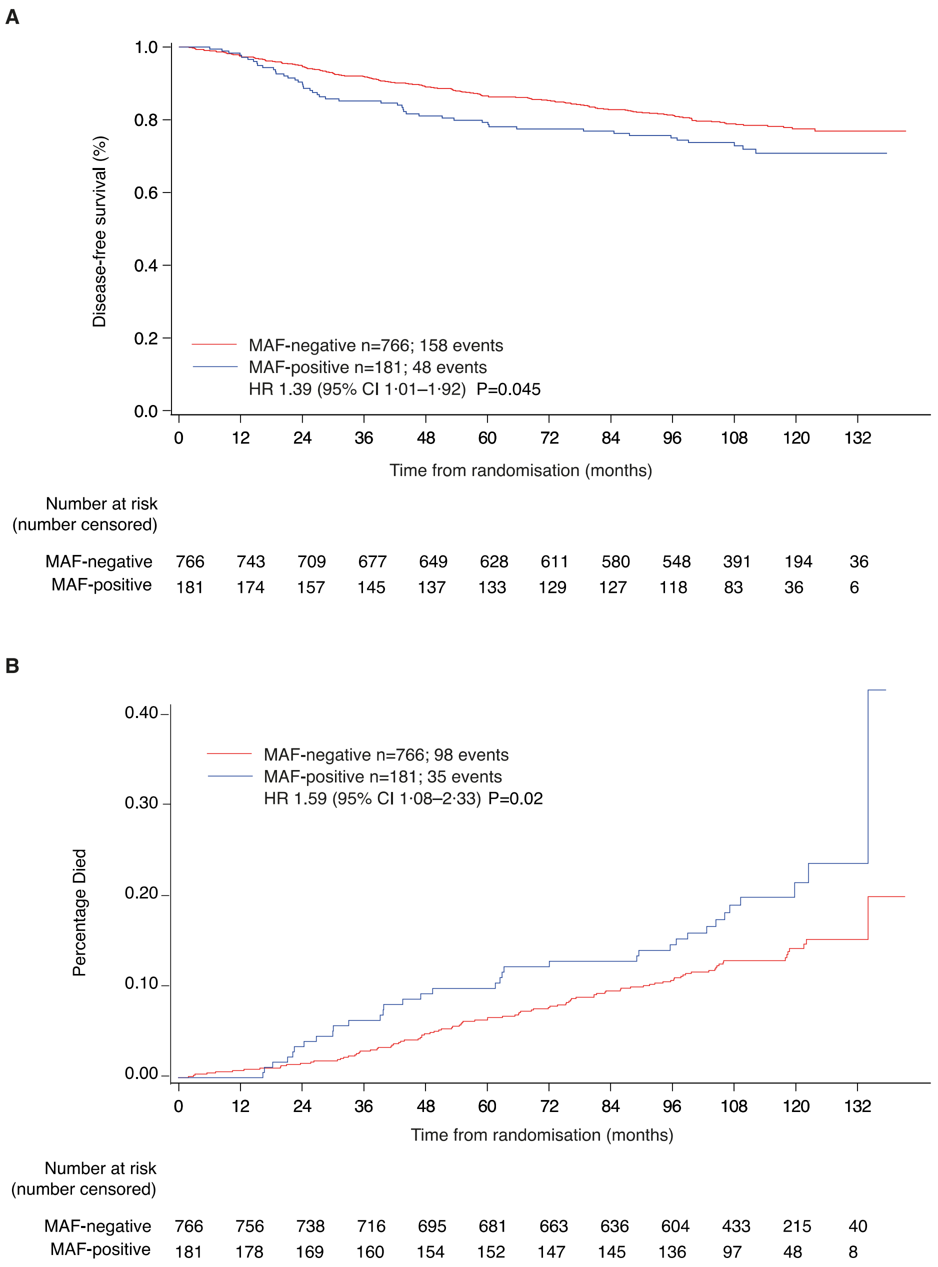

Supplementary Figure 2. MAF amplification prognostic capacity. A) Disease-free survival and B) Percentage died by *MAF* status in placebo treated patients Output at complete follow up from Cox multivariable model. HR=hazard ratio, CI=Confidence Interval
